# Single amino acid substitutions in the selectivity filter render *Nb*XIP1;1α aquaporin water permeable

**DOI:** 10.1186/s12870-017-1009-3

**Published:** 2017-03-09

**Authors:** Henry Ampah-Korsah, Yonathan Sonntag, Angelica Engfors, Andreas Kirscht, Per Kjellbom, Urban Johanson

**Affiliations:** Center for Molecular Protein Science, Department of Biochemistry and Structural Biology, Lund University, Box 124, SE-221 00 Lund, Sweden

**Keywords:** XIP, Homology modeling, Mutation, MIPs, Major Intrinsic Proteins, Boric acid

## Abstract

**Background:**

Aquaporins (AQPs) are integral membrane proteins that facilitate transport of water and/or other small neutral solutes across membranes in all forms of life. The X Intrinsic Proteins (XIPs) are the most recently recognized and the least characterized aquaporin subfamily in higher plants. XIP1s have been shown to be impermeable to water but permeable to boric acid, glycerol, hydrogen peroxide and urea. However, uncertainty regarding the determinants for selectivity and lack of an activity that is easy to quantify have hindered functional investigations. In an effort to resolve these issues, we set out to introduce water permeability in *Nicotiana benthamiana* XIP1;1α (*Nb*XIP1;1α), by exchanging amino acid residues of predicted alternative aromatic/arginine (ar/R) selectivity filters of *Nb*XIP1;1α for residues constituting the water permeable ar/R selectivity filter of *At*TIP2;1.

**Results:**

Here, we present functional results regarding the amino acid substitutions in the putative filters as well as deletions in loops C and D of *Nb*XIP1;1α. In addition, homology models were created based on the high resolution X-ray structure of *At*TIP2;1 to rationalize the functional properties of wild-type and mutant *Nb*XIP1;1α. Our results favour Thr 246 rather than Val 242 as the residue at the helix 5 position in the ar/R filter of *Nb*XIP1;1α and indicate that the pore is not occluded by the loops when heterologously expressed in *Pichia pastoris*. Moreover, our results show that a single amino acid substitution in helix 1 (L79G) or in helix 2 (I102H) is sufficient to render *Nb*XIP1;1α water permeable. Most of the functional results can be rationalized from the models based on a combination of aperture and hydrophobicity of the ar/R filter.

**Conclusion:**

The water permeable *Nb*XIP1;1α mutants imply that the heterologously expressed proteins are correctly folded and offer means to explore the structural and functional properties of *Nb*XIP1;1α. Our results support that Thr 246 is part of the ar/R filter. Furthermore, we suggest that a salt bridge to an acidic residue in helix 1, conserved among the XIPs in clade B, directs the orientation of the arginine in the ar/R selectivity filter and provides a novel approach to tune the selectivity of AQPs.

**Electronic supplementary material:**

The online version of this article (doi:10.1186/s12870-017-1009-3) contains supplementary material, which is available to authorized users.

## Background

Major Intrinsic Proteins (MIPs), commonly referred to as aquaporins (AQPs), constitute a large superfamily of channel proteins permeable to water and/or small uncharged solutes [1]. AQPs are found in all forms of life and are particularly in high abundance in plants [2–6]. There are seven aquaporin subfamilies in plants, namely: Plasma membrane Intrinsic Proteins (PIPs), Tonoplast Intrinsic Proteins (TIPs), Nodulin 26-like Intrinsic Proteins (NIPs), Small basic Intrinsic Proteins (SIPs), Glycerol facilitator-like Intrinsic Proteins (GIPs), Hybrid Intrinsic Proteins (HIPs) and X Intrinsic Proteins (XIPs) [2, 7, 8].

**Fig. 1:**
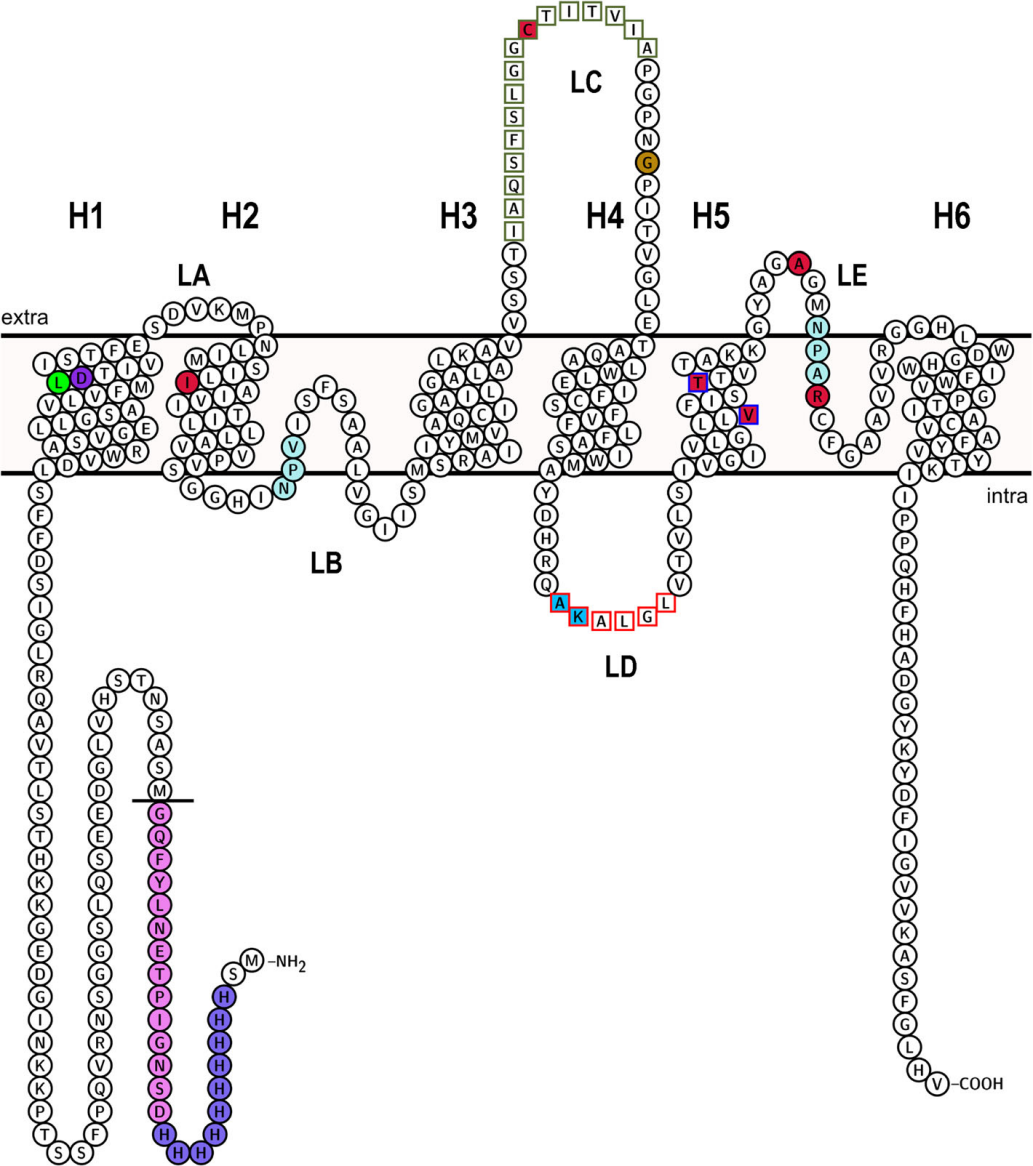
Topology of *Nb*XIP1;1αwt. The topology of the *Nb*XIP1;1αwt protein showing the amino acid residues in the aromatic/arginine (ar/R) selectivity filter and the mutated residues in the *Nb*XIP1;1α mutants. The two half helices in loops B and E are not shown in this representation. The N-terminal His_10_-tag and the TEV protease cleavage site are in medium *slate blue* and *violet* fills, respectively. The first amino acid residue, methionine, of the *Nb*XIP1;1αwt protein after the His_10_-tag and the TEV protease cleavage site has been underlined. The NPA aquaporin motifs are in *pale turquoise* fill. The ar/R filter residues I102 (H2^P^), C175 (LC^P^), V242/T246 (H5^P^), A257 (LE^P^) and R263 (HE^P^) are in *crimson* fill. Alternative residues, V242/T246, at the H5^P^ position in the ar/R filter are in *blue* square frame. The residue, G186, at the LC^P^ position in the models is in dark golden fill. L79 in helix 1 is in *lime* fill. Deleted residues (I165-A180) in the loop C of *Nb*XIP1;1α mutants are in *dark olive green* square frame. Deleted residues (A222-K223) in the loop D of *Nb*XIP1;1α/L79G/I102H/V242I mutants are in *deep sky blue* fill. Deleted residues (A222-L227) in the loop D of *Nb*XIP1;1α/L79G/I102H/T246I mutants are in *red* frame. D80 in helix 1 is in *blue violet* fill. The topology model was created in Protter [46]

The tetrameric aquaporin structure, wherein each monomer forms a functional pore, appears to be conserved among all AQPs. The monomer consists of six transmembrane helices (helix 1 – helix 6) connected by five loops (loop A – E), and both the N and C termini are located in the cytoplasm (Fig. 1). Loops B and E fold back into the membrane from opposite sides and form two short helices, denoted helix B and helix E, that jointly add a seventh α-helical transmembrane structure to the right-handed α-helical bundle of the monomer. These two half α-helices contain the asparagine-proline-alanine (NPA) aquaporin motif at their N-terminal ends that meet in the middle of the protein. Substrate permeability of AQPs is governed by two regions in the pore.

Firstly, the macro-dipoles of the two half-helices contribute to repulsion of positive charges at the conserved NPA region [9] and secondly, the variable aromatic arginine (ar/R) selectivity filter region modulates substrate specificity in AQPs by providing a substrate-specific aperture and specific interactions that fit the substrate [10]. This second region was originally thought to consist of four amino acid residues [11–14], however, the recently solved X-ray crystal structure of the *Arabidopsis thaliana* TIP2;1 aquaporin (*At*TIP2;1) at an atomic resolution of 1.18 Å revealed an extended ar/R filter with five amino acid residues at specific positions in the pore [14]. In addition to the initially identified positions in helices 2, 5, E (denoted H2^P^, H5^P^, HE^P^) as well as in loop E (LE^P^), a position in loop C (LC^P^) was uncovered by this structure. Furthermore, in the ammonia and water permeable *At*TIP2;1 the conserved arginine at position HE^P^ of the ar/R selectivity filter was found in a new spatial location stabilized by a hydrogen bond to a histidine at position H2^P^. Mutational studies of human AQP1 showed that it was possible to mimic the TIP2-like substrate profile [14], suggesting that a similar orientation of the arginine may be induced by mutations also in other AQPs.

XIPs are easy to distinguish from other AQPs due to their conserved LGGC and NPARC motifs in loop C and in helix E, respectively [7]. To date, XIPs have been found mostly in dicotyledonous plants, fungi and protozoa [5–8, 15–17]. Based on phylogenetic analyses plant XIPs are divided into four subgroups (I-IV) of which group I consists of XIPs from lower plants like moss and spikemoss [18]. The XIPs of eudicots are divided into clades A and B, corresponding to subgroups II and III + IV, respectively. It is clear that the XIP subfamily has gone through a relatively recent lineage specific expansion, as some groups with multiple paralogs are only found in closely related taxa [18]. In *Nicotiana benthamiana* there are, for example, three closely related genes, *NbXIP1;1–1;3,* of which *NbXIP1;1* and *1;2* are alternatively spliced [19]. Although purifying selection has been the main evolutionary force, it appears that plant XIPs have gone through periods of positive selection [18]. Thus the evolutionary history of plant XIPs indicate that the different clades of XIPs have different functions. According to sequence similarity and proposed substrate selectivity, XIPs were originally thought to be related to NIPs [7, 8]. However, in primitive plants a functional redundancy of XIPs and TIPs has been suggested based on the occurrence of a histidine at position H2^P^ of the selectivity filter [20]. Furthermore, some support for a deep orthology with HIPs, TIPs and animal AQP8s (all with a histidine at H2^P^) has been put forward based on phylogenetic studies [21]. Altogether this suggests that the structure of *At*TIP2;1 should be a suitable template for homology modeling of XIPs.

*At*TIP2;1 has been reported to be highly permeable to ammonia and water [14, 22–25]. On the other hand, XIPs were predicted and later found to be impermeable to water but permeable to boric acid, glycerol, urea, and hydrogen peroxide [7, 8, 26]. In a recent study, the amino acid residue at the helix two position (H2^P^) in the ar/R filter showed a significant divergence among XIPs [18], suggesting that the residue at this position may have a strong effect on the substrate specificity of XIPs.

XIPs deviate from other AQPs in several ways and this has made it difficult to align them, especially in loops and in the region corresponding to helix 5. Based on the conservation of a glycine residue in helix 5, two alternative alignments have been suggested, placing a valine or a threonine at the H5^P^ position of most dicot XIPs, whereof the former alignment is favoured by hydrophobicity plots and an apparent release of structural constraints [20]. However, the identity of the residue in position H5^P^ of the ar/R filter as well as the length of the flanking loops D and E are yet to be determined.

As mentioned above, the *XIP1;1* pre-RNA in *N. benthamiana* can be alternatively spliced resulting in two different *Nb*XIP1;1 proteins [19]. The *Nb*XIP1;1α protein studied here has a one amino acid shorter N-terminal region compared to the *Nb*XIP1;1β protein. In a recent study we demonstrated that the *Nb*XIP1;1α splice-variant is permeable to boric acid when expressed in the yeast *Pichia pastoris,* both by growth assays and when purified and reconstituted in proteoliposomes [19]. However, attempts to quantify boric acid permeability in yeast spheroplasts were unsuccessful (unpublished results). Based on the modest boric acid permeability of the reconstituted *Nb*XIP1;1α in proteoliposomes it was suggested that the protein was gated or only partially active. Interestingly, the heterologously expressed and purified protein was found to be phosphorylated at several positions in the N-terminal region, although the level of phosphorylation was not quantified [19]. It is conceivable that the pore is occluded by loop C or D since both are relatively long in the XIPs. In contrast to other subfamilies, XIPs and PIPs have a D loop of similar length which is thought-provoking as this loop is central in the gating of PIPs [27]. Depending on the exact alignment of helix 5, loop D in XIPs is either one amino acid residue shorter or three residues longer than in the PIPs.

Mutational studies of *Nb*XIP1;1α have been hampered by the lack of an activity that is easy to quantify directly in spheroplasts, omitting the time consuming steps of purifying and reconstituting each mutant protein, which is needed when reconstituting AQPs in proteoliposomes. In an effort to introduce water permeability in the *Nb*XIP1;1α and to gain in-depth understanding of the function of the different amino acids of the XIP selectivity filter, we set out to exchange the *Nb*XIP1;1α ar/R filter for the *At*TIP2;1 ar/R filter. In this study, we present functional results of amino acid substitutions in the filter as well as deletions in loops C and D of *Nb*XIP1;1α*.* Furthermore, we have created and analysed homology models based on the *At*TIP2;1 crystal structure to rationalize the functional properties of wild-type and mutant *Nb*XIP1;1α.

Our characterization of the heterologously expressed *Nb*XIP1;1α variants aims for a molecular understanding of the various XIP isoforms and of plant AQPs in general. Further studies in planta are required to elucidate the physiological role of the different clades of XIPs in the context of the whole plant.

## Methods

### Construction of *Nb*XIP1;1α mutants

According to the Thermo Scientific Phusion Site-Directed Mutagenesis Kit manual, 5′- phosphorylated primers were designed and used in polymerase chain reactions employing a modified pPICZB vector containing *Nb*XIP1;1α cDNA [19] as a template to generate pPICZB fragments harbouring the cDNA of *Nb*XIP1;1α mutants. The modified pPICZB confers a His_10_-tag and a TEV protease cleavage site to the N-terminus of the *Nb*XIP1;1α amino acid sequence. The primers used in the PCR and the resulting *Nb*XIP1;1α mutants are shown in Additional file 1: Table S1 and Table 2, respectively. The resulting PCR products were circularized, and transformed into *E. coli* strain XL1-Blue MRF’ and the constructs were verified by sequencing of the purified plasmids.

### Transformation of *Nb*XIP1;1α mutants into *Pichia pastoris*

The plasmids were transformed into wild-type *Pichia pastoris* X-33 cells by electroporation according to the EasySelect™ *Pichia* Expression kit manual [28]. The empty pPICZB was also transformed into *Pichia pastoris* X-33 cells as a negative control. Clones with potentially high copy numbers of *Nb*XIP1;1α mutants were selected on YPDS (1% (w/v) yeast extract, 1% (w/v) peptone, 2% (w/v) dextrose, 1 M sorbitol) and YPD (1% (w/v) yeast extract, 1% (w/v) peptone, 2% (w/v) dextrose) agar plates containing different concentrations of zeocin as published [19, 29].

### Small-scale expression

A small-scale expression screen was performed in order to analyze the expression levels in X-33 clones selected at the different antibiotic concentrations as described earlier [19]. In brief, *Nb*XIP1;1α mutant clones were cultured in 5 mL BMGY (1% (w/v) yeast extract, 2% (w/v) peptone, 100 mM potassium phosphate pH 6.0, 1.34% (w/v) yeast nitrogen base, 4 × 10^−5^% (w/v) biotin, 1% (v/v) glycerol) overnight to generate biomass. The *Pichia* cells were harvested and resuspended in 5 mL BMMY (1% (w/v) yeast extract, 2% (w/v) peptone, 100 mM potassium phosphate pH 6.0, 1.34% (w/v) yeast nitrogen base, 4 × 10^−5^% (w/v) biotin, 0.5% (v/v) methanol) to an optical density at 600 nm (OD_600_) of 1. Methanol was added to a final concentration of 0.5% (v/v) every 24 h to sustain protein induction. The cell cultures were incubated at 28 °C with continuous shaking at 245 rpm for 72 h. Cells corresponding to 40 OD_600_ units were harvested and lysed, by vortexing with glass beads, in 100 μL cold breaking buffer (50 mM NaPO_4_ pH 7.4, 1 mM EDTA, 5% (v/v) glycerol, 1 mM PMSF). The lysate was clarified by centrifugation and the supernatant, containing the crude cell extract was analyzed for *Nb*XIP1;1 content by western blot.

### Western blot analysis

The crude cell extracts were incubated in 3.33 × SDS loading buffer (250 mM Tris–HCl pH 6.8, 40% (v/v) glycerol, 8% (w/v) SDS, 2.4 M β-mercaptoethanol, 0.1% (w/v) bromophenol blue) for 30 min at room temperature. The proteins were separated on 12% SDS-PAGE gels and transferred onto polyvinylidene difluoride (PVDF) membranes (Millipore). His-tagged *Nb*XIP1;1α mutant proteins were visualized by probing with a monoclonal mouse 6xHis primary antibody (Clontech), using a horseradish peroxidase-conjugated polyclonal goat anti-mouse IgG as secondary antibody. Blots were developed by enhanced chemiluminiscence (ECL). For quantification of His-tagged *Nb*XIP1;1α mutant proteins, Syngene PXi touch instrument was used to scan the protein signals on the membrane (see Additional file 2: Figure S1). The scanned images were analyzed with the ImageJ software [30].

### Functional assay in *Pichia* spheroplasts

To test for water permeability in the *Nb*XIP1;1α mutants, *P. pastoris* X-33 spheroplasts expressing *Nb*XIP1;1α mutant proteins were prepared as previously described [19, 31]. Briefly, *Nb*XIP1;1α protein production was induced in the transformed *P. pastoris* cells in BMMY with a starting OD_600_ = 1, as described earlier. After 26 h of induction, the cells were harvested, re-suspended and incubated in TE-buffer (100 mM Tris–HCl, pH 8.0; 1 mM EDTA) and 0.5% β-mercaptoethanol for 1.5 h to destabilize the cell wall. The cells were then washed and equilibrated in 20 mM Tris–HCl pH 8, 1.2 M sorbitol to a final OD_600_ of 5. Equilibrated spheroplasts were challenged with a hyperosmolar solution (20 mM Tris–HCl pH 8, 1.8 M sorbitol) and the shrinkage upon mixture was observed by increased light scattering in a stopped-flow apparatus (SF-61 DX2 Double Mixing Stopped-flow System, Hi-Tech Scientific) at 500 nm. Kinetic Studio version 2.28 (TgK Scientific Limited) was used to calculate the rate constants after fitting the average of at least 15 traces to a single exponential equation. This functional assay was repeated three times and the standard deviation of the rate constant for water permeability was calculated. To be able to compare water permeability among the *Nb*XIP1;1α mutants, the rate constants were divided by the individual protein amounts estimated by western blot.

### Statistical analysis

Unpaired *t*-test assuming Gaussian distribution with Welch’s correction was used for the analysis of the data in GraphPad Prism [32].

### Homology modeling

Homology modeling of the *Nb*XIP1;1α was carried out with I-TASSER version 4.4 [33] using the *At*TIP2;1 structure (PDB ID 5I32) [14] as a template. Multiple sequence alignment of related AQPs used in the modeling was performed manually. Two alternative alignments of the helix 5 of *Nb*XIP1;1α were submitted to the I-TASSER server, including constraints for the IC**V**AR variant of the ar/R selectivity filter (one letter code of residues in H2^P^, LC^P^, **H5** ^**P**^, LE^P^, HE^P^). Point mutations were introduced in the wild-type *Nb*XIP1;1α (*Nb*XIP1;1αwt) model using Coot [34] and the mutated residue and immediate surroundings were subsequently energy minimized using NAMD [35]. The introduced H102 was singly protonated at Nδ. No attempt was made to model truncated loops or to further optimize the loops in the models. All models were supplemented with waters in the pore and subjected to geometry optimization using FG-MD [36]. Validation of model geometry was done using MolProbity [37]. The radius of the pore in the models was analysed by the program HOLE [38].

## Results

### Design of *Nb*XIP1;1α mutants

**Fig. 2:**
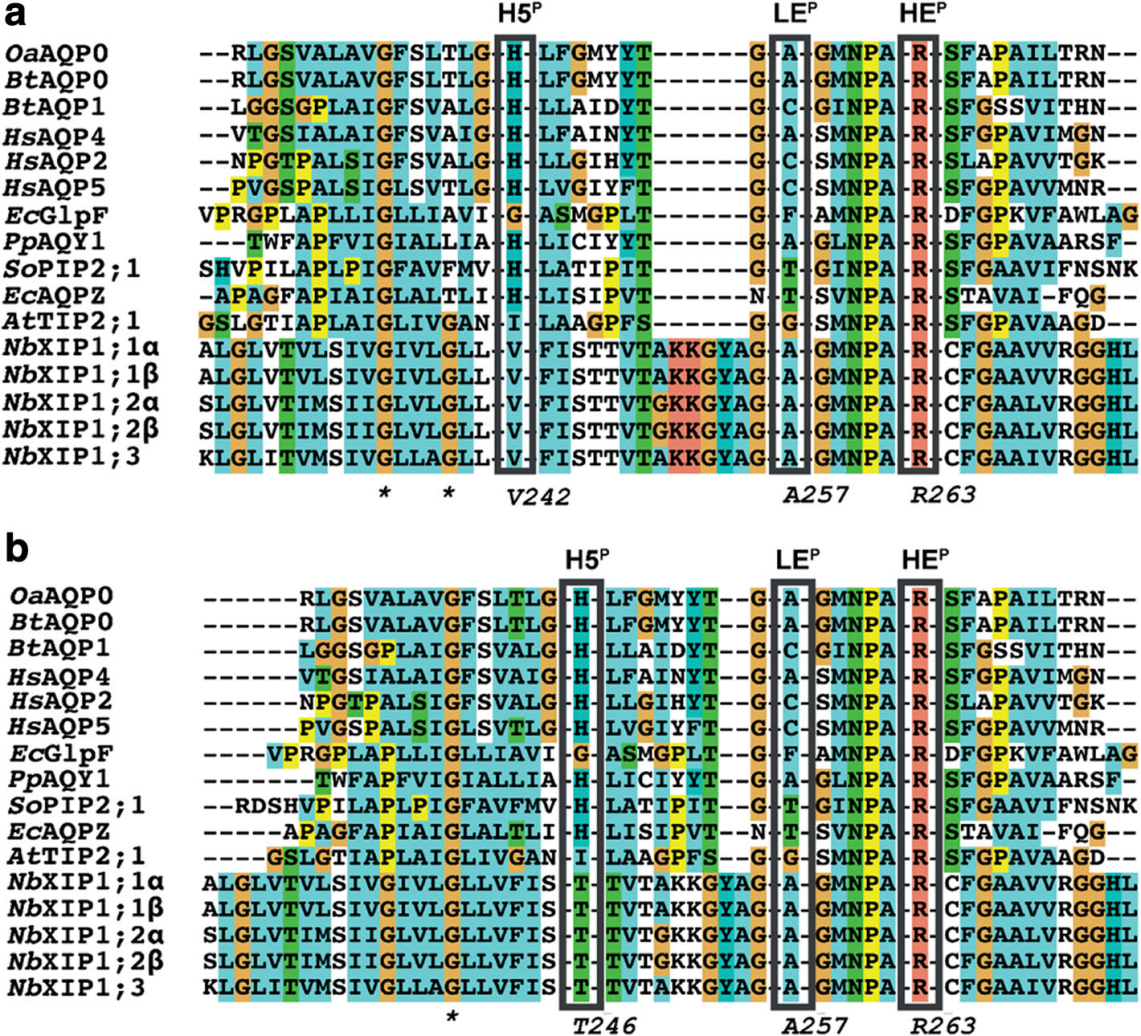
Alternative alignments of helix 5 in *Nb*XIP1s. Multiple sequence alignments showing two possible helix 5 alignments of *Nb*XIP1s with sequences of AQPs with solved crystal structures. The H5^P^, LE^P^ and HE^P^ positions representing the helix 5, loop E and helix E positions respectively of the aromatic arginine selectivity filter are shown in *black* boxes. **a** IC**V**AR alignment aligns the two glycines (*asterisk*) in the helix 5 of *Nb*XIP1s with the two glycines in the helix 5 of *At*TIP2;1. This alignment places valine 242 of *Nb*XIP1;1α at the H5^P^ position of the aromatic arginine selectivity filter. **b** IC**T**AR alignment aligns only one of the glycines in the helix 5 of *Nb*XIP1s with the conserved glycine in the helix 5 of the AQPs with solved structures. This alignment places threonine 246 of *Nb*XIP1;1α at the H5^P^ position of the aromatic arginine selectivity filter

Structurally guided multiple sequence alignment of *Nb*XIP1:1α to AQPs with structures deposited in the Protein Data Bank (PDB), resulted in two alternative alignments of residues in the ar/R selectivity filter of *Nb*XIP1;1α (Fig. 2, showing last three of the five residues in the filter). Thus, it could be comprised of isoleucine, cysteine, valine, alanine and arginine (IC**V**AR) or isoleucine, cysteine, threonine, alanine and arginine (IC**T**AR; putative residue at H5^P^ in bold) depending on how helix five was aligned. To introduce water permeability in *Nb*XIP1;1α, two putative selectivity filters of *Nb*XIP1;1α (I102, C175, **V242/T246**, A257, R263) were designed to mimic the *At*TIP2;1 filter (H63, H131, I185, G194, R200; Table 1).

**Table 1:** Aromatic arginine (ar/R) selectivity filter of *Nb*XIP1 isoforms, human and other plant aquaporin isoforms

MIPs	H2^P^	LC^P^	H5^P^	LE^P^	HE^P^
*Nb*XIP1;1s	I	C^a^	V/T^b^	A	R
*Nb*XIP1;2s	A	C^a^	V/T^b^	A	R
*Nb*XIP1;3	I	C^a^	V/T^b^	A	R
*At*NIP1s, 2;1, 4s	W	S/T	V	A	R
*At*NIP3;1	W	T	I	A	R
*At*NIP5;1	A	T	I	G	R
*At*NIP6;1	A	T	I	A	R
*At*NIP7;1	A	T	V	G	R
*At*TIP1s	H	F	I	A	V
*At*TIP2s	H	H	I	G	R
*At*TIP3s	H	F	I	A	R
*At*TIP5;1	N	Y	V	G	C
*Hs*AQP8	H	F	I	G	R
*At*PIPs	F	N	H	T	R

^a^Not supported by models in this article. However, this part of the model is less reliable due to little sequence similarity with structural template and no effort was made to model the loop regions

^b^Most likely T (Thr) according to the results in this article

Due to the uncertainty in the alignment of loop C and the conservation of the residues at LE^P^ and HE^P^ only the two positions of the filter corresponding to H2^P^ and H5^P^ were considered. However, an L79G substitution in helix 1, that is not part of the selectivity filter, was added since a small amino acid residue is conserved at this position in AQPs with an aromatic amino acid residue in H2^P^ and this appears to be a prerequisite to accommodate the introduced histidine at H2^P^ in a TIP2;1-like orientation. Thus, two principal *Nb*XIP1;1α mutants were constructed; *Nb*XIP1;1αL79G/I102H/**V242I** and *Nb*XIP1;1αL79G/I102H/**T246I** (Table 2; differing mutations putatively in H5^P^ in bold). To exclude the possibility of poor water permeability due to occlusion of the pore by the long loop C and loop D, the corresponding *Nb*XIP1;1α mutants with deletions in loop C and/or loop D were also generated. The truncations were guided by the two alternative alignments to achieve the same length of these loops as found in *At*TIP2;1. To further investigate the contribution of the individual substitutions, six additional mutants (Table 2) were subsequently constructed based on the functional results presented below.

**Table 2:** List of *Nb*XIP1:1α mutants

*Nb*XIP1;1α mutant	Mutation	Position
*Nb*XIP1;1αL79G	L79G	Helix 1
*Nb*XIP1;1αI102H	I102H	Helix 2
*Nb*XIP1;1α**V242I**	V242I	Helix 5
*Nb*XIP1;1αL79G/I102H	L79G, I102H	Helix 1, Helix 2
*Nb*XIP1;1αL79G/**V242I**	L79G, V242I	Helix 1, Helix 5
*Nb*XIP1;1αI102H/**V242I**	I102H, V242I	Helix 2, Helix 5
*Nb*XIP1;1αL79G/I102H/**V242I**	L79G, I102H, V242I	Helix 1, Helix 2, Helix 5
*Nb*XIP1;1αL79G/I102H/**V242I**/ΔC	L79G, I102H, V242I, ΔI166-A181	Helix 1, Helix 2, Helix 5, Loop C
*Nb*XIP1;1αL79G/I102H/**V242I**/ΔD	L79G, I102H, V242I, ΔA222-K223	Helix 1, Helix 2, Helix 5, Loop D
*Nb*XIP1;1αL79G/I102H/**V242I**/ΔC/ΔD	L79G, I102H, V242I, ΔI166-A181, ΔA222-K223	Helix 1, Helix 2, Helix 5, Loop C, Loop D
*Nb*XIP1;1αL79G/I102H/**T246I**	L79G, I102H, T246I	Helix 1, Helix 2, Helix 5
*Nb*XIP1;1αL79G/I102H/**T246I**/ΔC	L79G, I102H, T246I, ΔI166-A181	Helix 1, Helix 2, Helix 5, Loop C
*Nb*XIP1;1αL79G/I102H/**T246I**/ΔD	L79G, I102H, T246I, ΔA222-L227	Helix 1, Helix 2, Helix 5, Loop D

### Heterologous expression of *Nb*XIP1;1α mutants in *Pichia pastoris*

**Fig. 3:**
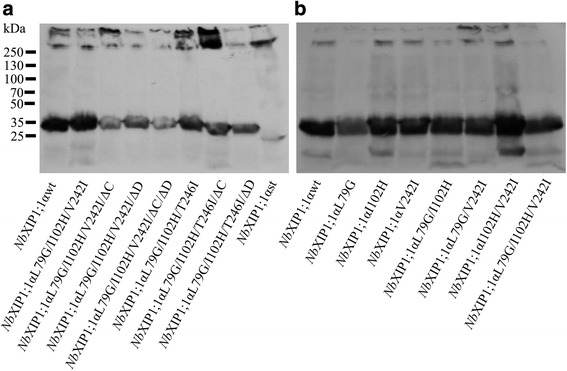
Expression of N-terminally His-tagged *Nb*XIP1;1α mutants in *P. pastoris*. Western blots showing the expression levels of N-terminally His-tagged *Nb*XIP1;1 mutants in *P. pastoris* X-33 clones. Blots were developed on photographic films by enhanced chemiluminiscence. See Additional file 2: Figure S1 for the estimation of protein amounts. **a** First set of *Nb*XIP1;1α mutants. *Nb*XIP1;1αst is an N-terminally truncated construct of *Nb*XIP1;1α with a fully deleted N-terminal region used as positive control for the western blot. **b** Second set of *Nb*XIP1;1α mutants

Mutant proteins of the *Nb*XIP1;1α splice-variant were successfully expressed in *P. pastoris*, although at different levels. Hence, to obtain adequate protein amounts detectable by western blot, protein induction in *P. pastoris* was extended and more cells were used compared to the standard protocol [19]. After this modification, crude cell extracts prepared from *P. pastoris* cells expressing the mutant proteins had appreciable amounts of His-tagged *Nb*XIP1;1α mutant proteins, as shown in Fig. 3a and b. In general, protein expression was lower for constructs containing deletions and higher in cells expressing *Nb*XIP1;1αL79G/I102H or *Nb*XIP1;1αI102H/**V242I** (Additional file 2: Figure S1, Additional file 3: Table S2, Additional file 4: Table S3).

### The triple mutant *Nb*XIP1;1αL79G/I102H/V242I is permeable to water

**Fig. 4:**
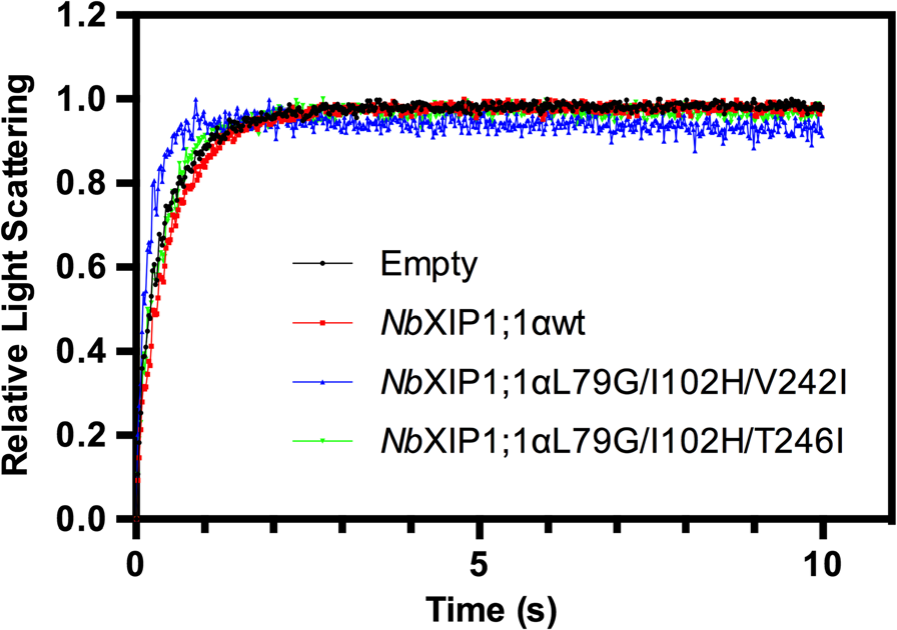
Water permeability in *P. pastoris* spheroplasts. Stopped-flow traces showing kinetics of osmotic water permeability in spheroplasts with empty pPICZB plasmid (*black*), spheroplasts expressing *Nb*XIP1;1αwt (*red*), spheroplasts expressing *Nb*XIP1;1αL79G/I102H/**V242I** (*blue*) and spheroplasts expressing *Nb*XIP1;1αL79G/I102H/**T246I** (*green*). The traces (mean of at least 15 traces) were fitted to single exponential equations. The mean rate constants ± standard deviations for the fitted curves were: 2.19 ± 0.25 s^−1^ (Empty); 2.20 ± 0.42 s^−1^ (*Nb*XIP1;1αwt); 5.52 ± 1.21 s^−1^ (*Nb*XIP1;1αL79G/I102H/**V242I**) and 2.84 ± 0.48 s^−1^ (*Nb*XIP1;1αL79G/I102H/**T246I**)

To compare the water permeability of the yeast spheroplasts prepared from induced cells expressing the various constructs, a stopped-flow spectrometric assay was employed. As shown in Fig. 4, there was little or no difference in the rate of shrinkage between the control *P. pastoris* spheroplasts with the empty vector pPICZB and the spheroplasts overexpressing *Nb*XIP1;1αwt, which suggests poor or no water permeability of the protein. The rate of shrinkage of spheroplasts expressing the mutant *Nb*XIP1;1αL79G/I102H/**T246I** was only slightly increased whereas expression of *Nb*XIP1;1αL79G/I102H/**V242I** resulted in a more than two-fold higher rate. The mean rate constants and standard deviations for the fitted curves were: 2.19 ± 0.25 s^−1^ (empty pPICZB plasmid), 2.20 ± 0.42 s^−1^ (*Nb*XIP1;1αwt), 2.84 ± 0.48 s^−1^ (*Nb*XIP1;1αL79G/I102H/**T246I**) and 5.52 ± 1.21 s^−1^ (*Nb*XIP1;1αL79G/I102H/**V242I**). To compensate for differences in expression, the background corrected rates were related to the protein levels to obtain a specific activity in arbitrary units. The estimated protein amounts, rate constants and specific activities for the first set of *Nb*XIP1;1α mutants are shown in Additional file 3: Table S2.

**Fig. 5:**
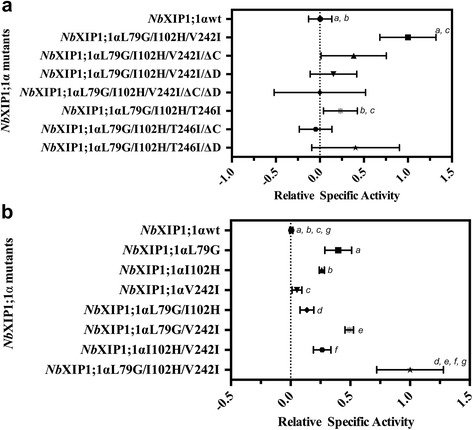
Relative specific activities of *Nb*XIP1;1α mutants for water permeability in *P. pastoris* spheroplasts. The specific activities of the individual *Nb*XIP1;1α mutants and the wild-type *Nb*XIP1;1α (*Nb*XIP1;1αwt) were normalized to the specific activity of the *Nb*XIP1;1αL79G/I102H/**V242I** mutant. The background corrected rate constants obtained from the osmotic water permeability stopped flow spectroscopy assay were divided by the individual protein amounts estimated by western blot to obtain the specific activities. **a** First set of *Nb*XIP1;1α mutants. *Nb*XIP1;1αL79G/I102H/**V242I**, *Nb*XIP1;1αL79G/I102H/**V242I**/ΔC, *Nb*XIP1;1αL79G/I102H/**V242I**/ΔD and *Nb*XIP1;1αL79G/I102H/**V242I**/ΔC/ΔD were designed based on the IC**V**AR alignment of *Nb*XIPs helix 5 while *Nb*XIP1;1αL79G/I102H/**T246I**, *Nb*XIP1;1αL79G/I102H/**T246I**/ΔC and *Nb*XIP1;1αL79G/I102H/**T246I**/ΔD were designed based on the IC**T**AR alignment. *a* and *c* indicate statistical significant differences (*P* < 0.05), whereas *b* is not significant different. See Additional file 5: Table S4. ΔC and ΔD indicate truncations in loop C and loop D. **b** Second set of *Nb*XIP1;1α mutants. Comparisons marked *a* and *b*, *d*, *f*, *g* indicate statistical significant differences at two different levels (*P* < 0.0005 and *P* < 0.05, respectively) while differences between pairs marked *c* and *e* are not significant. See Additional file 6: Table S5

Considering the average specific activities, the *Nb*XIP1;1αL79G/I102H/**V242I** triple mutant was almost 300-fold more permeable to water than the *Nb*XIP1;1αwt. Although there is considerable variation between replicates, the corresponding relative specific rates, setting the specific rate of *Nb*XIP1;1αL79G/I102H/**V242I** as unity, are significantly different as shown in Fig. 5 and Additional file 5: Table S4 (*P* < 0.05). In contrast, the relative specific rate of *P. pastoris* spheroplasts expressing *Nb*XIP1;1αL79G/I102H/**T246I** was not significantly different from that of the *Nb*XIP1;1αwt spheroplasts. However, the relative specific rate of *P. pastoris* spheroplasts expressing *Nb*XIP1;1αL79G/I102H/**T246I** was significantly lower than that of the spheroplasts with *Nb*XIP1;1αL79G/I102H/**V242I**.

To eliminate the possibility of the C and D loops blocking the pore, *Nb*XIP1;1αL79G/I102H/**V242I** and *Nb*XIP1;1αL79G/I102H/**T246I** mutants with loop C and/or loop D truncations were engineered. However, as shown in Fig. 5a, truncating the loops did not significantly improve the relative specific rates, as *P. pastoris* spheroplasts overexpressing *Nb*XIP1;1αL79G/I102H/**V242I**/ΔC, *Nb*XIP1;1αL79G/I102H/**V242I**/ΔD, *Nb*XIP1;1αL79G/I102H/**V242I**/ΔC/ΔD, *Nb*XIP1;1αL79G/I102H/**T246I**/ΔC or *Nb*XIP1;1αL79G/I102H/**T246I**/ΔD showed large experimental variation with averages not significantly different from the wild type or the *Nb*XIP1;1αL79G/I102H/**T246I** mutant without the loop deletions.

### Single substitutions L79G and I102H are sufficient to render *Nb*XIP1;1α water permeable

To ascertain whether all three amino acid substitutions in the *Nb*XIP1;1αL79G/I102H/**V242I** mutant are required to induce water permeability in *Nb*XIP1;1α, six additional *Nb*XIP1;1α mutants were constructed as shown in Table 2. The estimated protein amounts, rate constants and specific activities for *Nb*XIP1;1αwt, *Nb*XIP1;1αL79G/I102H/**V242I** and the second set of *Nb*XIP1;1α mutants were determined in the same way as for the first set and are reported in Additional file 4: Table S3. As displayed in Fig. 5b, when relating the specific rates to the triple mutant, all the other mutants showed lower specific rates that were significantly different from the triple mutant, except for *Nb*XIP1;1αL79G/**V242I** (Additional file 6: Table S5). Still, all except spheroplasts overexpressing *Nb*XIP1;1α**V242I** and *Nb*XIP1;1αL79G/I102H, showed significantly higher relative specific rates than the *Nb*XIP1;1αwt spheroplasts. Thus, even the single substitutions L79G and I102H are individually sufficient to increase the water permeability of the *Nb*XIP1;1α.

### Homology modeling and pore diameter

The wild-type *Nb*XIP1;1α (*Nb*XIP1;1αwt) was modeled using the *At*TIP2;1 structure as a template. Even though two alternative alignments (denoted IC**V**AR and IC**T**AR; Fig. 2) were submitted to I-TASSER, only the IC**T**AR variant could be modeled using this approach. Regardless of which initial alignment that was used, similar *Nb*XIP1;1α models were obtained with Thr 246 placed at the H5^P^ position in the ar/R filter. All attempts to force the I-TASSER modeler with different constraints to place Val 242 at the H5^P^ position proved futile, as it was energetically more favorable to place Thr 246 at the H5^P^ position instead.

**Fig. 6:**
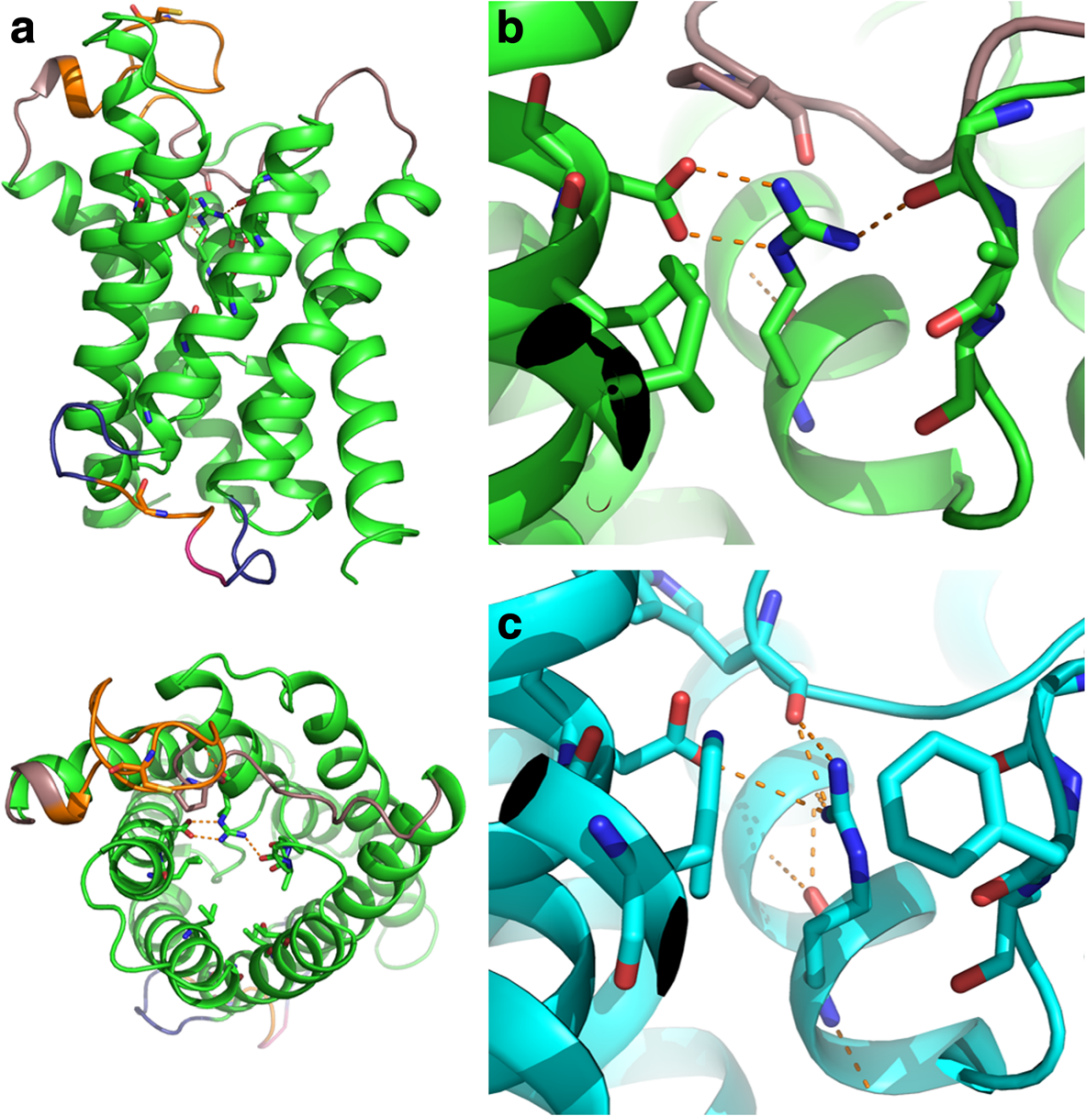
Cartoon representation of the homology model of *Nb*XIP1;1αwt and the structure of *Pf*AQP. The high resolution X-ray structure (PDB 5I32) of *At*TIP2;1 was used as template to model *Nb*XIP1;1αwt [14]. **a** Side view (*upper panel*) and top view (*below*) of *Nb*XIP1;1αwt model, showing the general aquaporin monomeric fold with 6 transmembrane helices and 2 half helices with interconnecting loops. The deletions in loop C (*orange at the top*) and loop D (*short: pink, long: pink + orange*) are also indicated. **b** Close-up of the salt-bridge between arginine (R263) of the ar/R selectivity filter and aspartate (D80) in helix 1 in the model. Except in the *Nb*XIP1;1αL79G/**V242I** model, the arginine (R263) also forms hydrogen bonds to the carbonyls of the backbone in loop E. This seems to be valid for all but one model of *Nb*XIP1;1α in this study **c**. In the *Pf*AQP (PDB ID 3CO2), the arginine (R196) at position HE^P^ in the ar/R filter interacts with an acidic residue (E28) in a corresponding position, but in contrast to the XIP-models the arginine of *Pf*AQP also forms hydrogen bonds to a carbonyl (W124) of the loop C backbone [45]

Overall the models are consistent with the general AQP structure and none of the loops appear to block the pore (Fig. 6a). Contrary to our expectation none of the models placed the highly conserved Cys 175 at the LC^P^ position, instead the much less conserved Gly 186 was found at this position in all the models. It should however be noted that the fold and position of the long C and D loops are unreliable since no extra effort was made to optimize these regions neither in the models of the monomer nor in a tetramer.

An unexpected result of the modeling is the novel orientation of the arginine (Arg 263) of the ar/R selectivity filter. In all the models of the *Nb*XIP1;1αwt and the mutants, Arg 263 forms a salt bridge to Asp 80 in helix 1 (Fig. 6b). Furthermore, the arginine makes hydrogen bonds to carbonyls of the backbone in loop E in all models except *Nb*XIP1;1αL79G/**V242I**, in which it instead interacts with a carbonyl of the loop C backbone. As a result of the salt bridge to Asp 80 no hydrogen bonds are formed between the arginine and the introduced histidine at H2^P^. In the model of *Nb*XIP1;1αL79G/I102H/**T246I** a TIP2-like orientation is adopted by the histidine but this is not sufficient to reorient the arginine to a similar position as found in the structure of *At*TIP2;1.

**Fig. 7:**
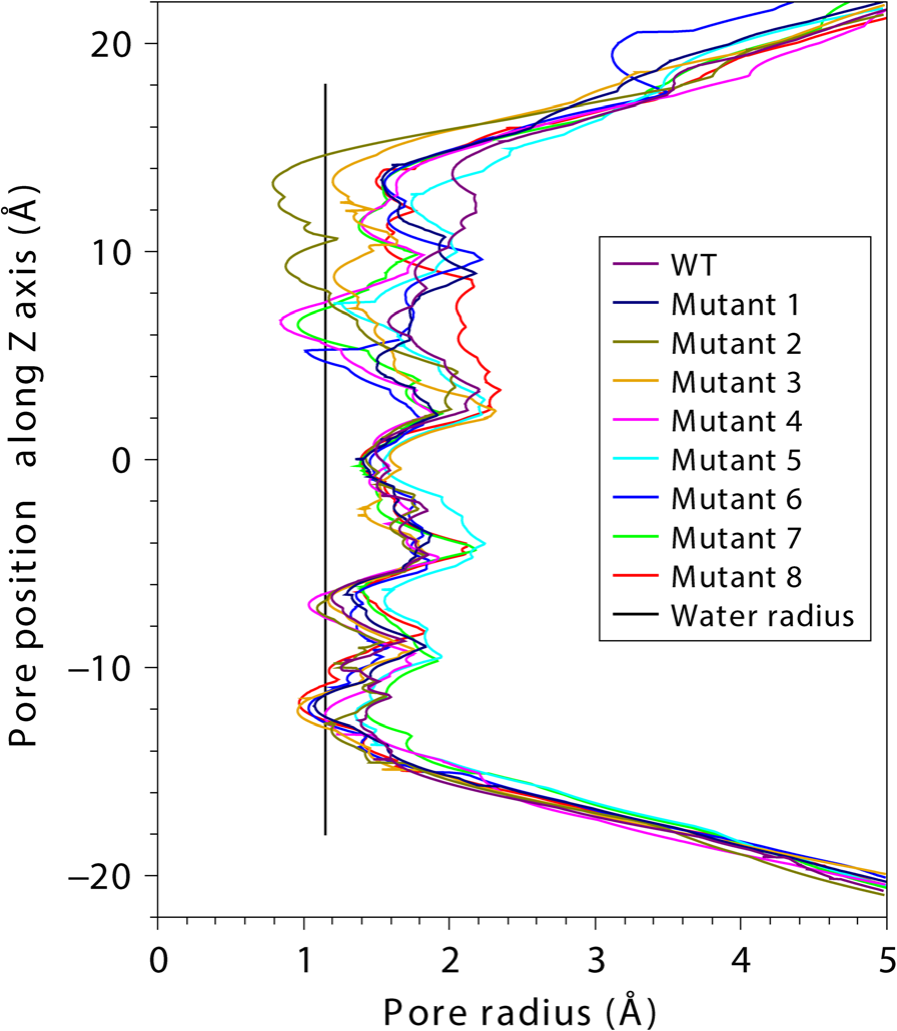
Estimation of the radius of the pore in *Nb*XIP1;1αwt and *Nb*XIP1;1α mutants. WT (*Nb*XIP1;1αwt), Mutant 1 (*Nb*XIP1;1αL79G/I102H/**V242I**), Mutant 2 (*Nb*XIP1;1αL79G/I102H/**T246I**), Mutant 3 (*Nb*XIP1;1αL79G), Mutant 4 (*Nb*XIP1;1αI102H), Mutant 5 (*Nb*XIP1;1α**V242I**), Mutant 6 (*Nb*XIP1;1αL79G/I102H), Mutant 7 (*Nb*XIP1;1αI102H/**V242I**) and Mutant 8 (*Nb*XIP1;1αL79G/**V242I**). The program HOLE [38] was used to estimate the radius of the pore in the models. See also Additional file 7: Figure S2 in the supplementary information

The program HOLE [38] was used to estimate the radius of the pore in the models of *Nb*XIP1;1αwt and the mutants (Fig. 7, Additional file 7: Figure S2). The result suggests that the pore of *Nb*XIP1;1αL79G/I102H/**T246I** is most narrow followed by *Nb*XIP1;1αI102H and *Nb*XIP1;1αI102H/**V242I**, whereas the pore of the *Nb*XIP1;1αwt and *Nb*XIP1;1αL79G/**V242I** appears less restricted. The constriction in *Nb*XIP1;1αL79G/I102H/**T246I** is indirectly formed by T246I since the isoleucine by steric hindrance and its hydrophobicity directs His 102 to the TIP2-like orientation at the same time as it is changing the location of loop E by interactions with Ala 257. In both *Nb*XIP1;1αI102H and *Nb*XIP1;1αI102H/**V242I** the pore radius is limited by the histidine and Leu 79.

## Discussion

The use of the *Pichia pastoris* expression system for the study of membrane proteins, especially AQPs, has gained a lot of interest in the past two decades. This is because, as a eukaryote, *Pichia* has the machinery to correctly express and fold eukaryotic proteins [29, 39–41]. All the 13 human AQPs and a number of plant AQPs have been studied in *P. pastoris* [19, 29, 42, 43]. As expected from earlier studies of the *Nb*XIP1;1αwt protein [19], expression of all the *Nb*XIP1;1α mutant proteins in *P. pastoris* was successful. The observed differences in the expression levels of the mutants could, however, be due to differences in gene dosage of the construct encoding the mutant protein in the various *Pichia* clones as shown previously by Nordén et al. [29].

With the exception of the loop truncations which could potentially alter the overall protein fold and the stability of the protein, the mutations were not expected to introduce any adverse effect on the overall protein structure, as the substitutions constitute subtle changes in the pore of *Nb*XIP1;1α. Since *Nb*XIP1;1αwt was found in the *P. pastoris* plasma membrane in a previous study [19], it is likely that the *Nb*XIP1:1α mutants are also localized to the plasma membrane of *P. pastoris* and therefore available for functional studies in spheroplasts.

In the present study we made an attempt to introduce water permeability in *Nb*XIP1:1α in order to facilitate functional studies and to probe our understanding of the functional determinants in *Nb*XIP1:1α, and this was indeed accomplished. The water permeable mutants as such support the idea that the overall AQP fold and trafficking to the plasma membrane is preserved in mutants harbouring subtle changes and allows different properties of *Nb*XIP1:1α, like a putative gating, to be explored in a constructive way. In the same manner, predicted interactions in the ar/R filter region may be investigated by mutations to get a deeper understanding of how the selectivity can be tuned, which may be valid also for other isoforms.

Aquaporin channel gating involving loop D has been reported in the plant aquaporin *So*PIP2;1 [27]. As shown by the truncated mutants in spheroplasts assay, we found no support for our concerns that the long loop C and/or loop D could be occluding the pore. Conversely, the inability of the *Nb*XIP1;1αL79G/I102H/**V242I** mutants with shortened loop C and/or D to facilitate the transport of water indicate non-functional conformations adopted by the mutants, possibly blocked by the truncated loops. It appears that the D loop is rather sensitive to mutations, as the deletion in this particular construct is only two amino acid residues long. However, it is possible that the folding in the membrane is compromised since the deletion removes a positively charged residue from the cytosolic side of the protein [44]*.*

The stopped-flow spectrometric analysis of the water permeability of the *Nb*XIP1;1α mutants in *Pichia* spheroplasts and the homology modeling of the *Nb*XIP1;1α mutants with *At*TIP2;1 as the template were performed concomitantly. Although it appeared from the initial mutational studies that we had succeeded in introducing water permeability by three TIP2-like substitutions in the ar/R selectivity filter, a more reasonable interpretation of our result is that V242I is a neutral mutation located outside the filter whereas T246I is positioned at H5^P^ and not compatible with L79G/I102H and water permeability, favoring the second alternative alignment (Fig. 2b). Thus, results from both the experimental data and from the *in-silico* modeling support the idea that Thr 246 and not Val 242 is the amino acid residue that resides at the H5^P^ position in the *Nb*XIP1;1α ar/R filter. However, further studies including X-ray crystallographic structural determination of *Nb*XIP1;1s are needed to definitively confirm this.

The *Nb*XIP1;1αwt was impermeable to water in this study just as *Pt*XIP1;1 and *Nt*XIP1;1 could not facilitate the transport of water in *Xenopus laevis* oocytes [8, 26]. The inability of XIP1;1s to facilitate the transport of water has been largely attributed to the hydrophobic nature of their selectivity filters [7, 8]. This idea was further supported by the finding that *Nt*XIP1;1s were permeable not only to glycerol, but also to urea and boric acid [8]. It is not immediately clear from our model why *Nb*XIP1;1αwt is not permeable to water. As the pore appears to be wide enough for water to pass, we assume that the presence of the hydrophobic residues (Ile 102 and Ala 257 at H2^P^ and LE^P^, respectively) in the ar/R filter prevent the hydrogen bonding network needed for water permeability in the *Nb*XIP1;1αwt.

The models of the *Nb*XIP1;1αL79G/I102H/**V242I** and *Nb*XIP1;1αL79G/I102H/**T246I** mutants revealed that the idea of instituting minimal changes in the *Nb*XIP1;1α ar/R filter, to mimic the *At*TIP2;1 ar/R filter in order to facilitate water permeability in *Nb*XIP1;1α, was too simplistic. Additional substitutions seem to be needed to achieve this goal, especially since the residues at the LC^P^ and H5^P^ positions are not precisely known due to alternative alignment possibilities. In particular, the salt bridge between arginine in the ar/R filter and Asp 80 is of interest and relevant in other isoforms as well. Interestingly, a similar interaction between the arginine and an acidic residue at the corresponding position to Asp 80 is found in the structure of *Pf*AQP, a glycerol facilitator of *Plasmodium falciparum* [45] (Fig. 6c). We note that an acidic residue at this position is not conserved among all groups of XIPs, e.g. in the XIPs of moss and spikemoss (XIP I; Table 3) that have a histidine or a glutamine in H2^P^, both of which may form a TIP2-like interaction with the arginine of the selectivity filter. Other XIPs lack both the salt bridge and a putative TIP2-like interaction between residues in H2^P^ and HE^P^, suggesting that there are at least three different orientations of the arginine in HE^P^ that are potentially tweaking the selectivity in the diverged phylogenetic groups of XIPs. There is also support for a similar modification of selectivity in TIP subgroups (Table 3). Both the TIP3s of angiosperms and the TIP1s of gymnosperms have a glutamate at the position that is aligning to Asp 80 in *Nb*XIP1;1α. Interestingly, within the TIP1 sister clade to the TIP3s, the monocot/dicot TIP1s which all lack the arginine are also missing an acidic residue corresponding to Asp 80.

**Table 3:** The residues corresponding to Asp 80 of *Nb*XIP1;1 and the residues in four of the positions in the ar/R selectivity filter

AQP	D80 (H1)^a^	H2^P^	H5^P^	LE^P^	HE^P^	Reference
XIP-I	I	H/Q^b^	G/A/Q	A/T	R	^c^
XIP-II, Clade A	T/S	V/I/G	I/V/T/S	V/A	R	^c^
XIP-III, Clade B	D	V/I/A	T/S	V/A	R	^c^
XIP-IV, Clade B	D	I/L/A	T	A/V	R	^c^
TIP1s, Gymnosperm	E	H^b^	I	A	R	^d^
TIP1s, Mono/dicot	Q/S	H	I	A	V	^d^
TIP3s, Mono/dicots	E	H^b^	I/M	A	R	^d^
*Pf*AQP	E	W	G	F	R	^e^

^a^The acidic residue corresponding to Asp 80 in helix 1 (H1) of *Nb*XIP1;1 is conserved in XIPs of clade B. Acidic residues at this position may form a salt bridge to arginine in HE^P^; ^b^XIP-Is and some TIP1s as well as TIP3s are likely to have a TIP2-like H2^P^ polar interaction with the arginine at HE^P^;^c^ [18]; ^d^ [20]; ^e^ [45]

Contrary to our naïve expectations, only the ar/R filter in the *Nb*XIP1;1αL79G/I102H/**T246I** mutant model resembled the *At*TIP2;1 ar/R filter, although it was the *Nb*XIP1;1αL79G/I102H/**V242I** mutant that was permeable to water. The latter could not only be attributed to the introduction of His 102 at the H2^P^ position to increase the possibility of hydrogen bonding but also to the introduction of Gly 79 which makes room for water permeability in the *Nb*XIP1;1αL79G/I102H/**V242I**. It was assumed that the V242I substitution in *Nb*XIP1;1αL79G/I102H/**V242I** did not influence water permeability, since according to the model, it is located deep in the pore and does not form part of the ar/R filter. It is noteworthy that the Thr 246 was left untouched in the *Nb*XIP1;1αL79G/I102H/**V242I** mutant and hence could provide hydrogen bonding possibilities to pore waters permeating the channel. The inability of the *Nb*XIP1;1αL79G/I102H/**T246I** mutant to facilitate water permeability may therefore be attributed to the lack of Thr 246 as well as the small aperture indirectly caused by the isoleucine via His 102 and Ala 257 as mentioned in the result section.

With the exception of the *Nb*XIP1;1α**V242I** mutant, all the single mutants engineered to investigate the individual contribution of the substitutions in the *Nb*XIP1;1αL79G/I102H/**V242I** triple mutant were more permeable to water than *Nb*XIP1;1αwt. As mentioned, it is not surprising that the *Nb*XIP1;1α**V242I** mutant had poor or no water permeability as according to the models the *Nb*XIP1;1α**V242I** ar/R filter is identical to that of the *Nb*XIP1;1αwt. Consistently, the substitution V242I appears neutral also when combined with mutation L79G or I102H separately. However, it is intriguing that the V242I substitution still seems crucial for water permeability in the triple mutant *Nb*XIP1;1αL79G/I102H/**V242I**, since the double mutant *Nb*XIP1;1αL79G/I102H has a lower relative specific rate, which is not significantly different from that of *Nb*XIP1;1αwt. We cannot explain this result based the models and it is possible that other factors like a proper folding of the protein and insertion in the plasma membrane come into play here, but from the recorded protein levels there is no clear pattern indicative of stability issues.

According to a recent study of the ar/R filter residues of XIPs, the residue at the H2^P^ position varied significantly between XIP isoforms [18] and as such the residues at this position, could influence substrate specificity in XIPs. Our results corroborate the proposition of Ile 102 in the H2^P^ position as the I102H substitution rendered *Nb*XIP1;1α water permeable. The model of the *Nb*XIP1;1αI102H mutant indicates that His 102 at the H2^P^ position in the ar/R filter can contribute to the hydrogen bond network in the pore. However, the extended hydrogen bonding network might stabilize water molecules, making them less likely to shift position through the pore. This could be the reason that the water permeability in the *Nb*XIP1;1αI102H mutant was not as high as in the *Nb*XIP1;1αL79G/I102H/**V242I** mutant, where additional substitutions increase the radius of the pore.

Our results suggest that in addition to the five amino acid residues of the ar/R filter, other residues, especially those lining the pore, influence substrate specificity in *Nb*XIP1;1α. Nonetheless, it was quite striking that the single amino acid substitution L79G in helix 1 made the *Nb*XIP1;1αL79G mutant permeable to water. Superimposing the *Nb*XIP1;1αL79G model on the *Nb*XIP1;1αwt model revealed that Arg 263 was shifted in position in the *Nb*XIP1;1αL79G mutant due to lack of steric hindrance as a result of the L79G substitution. It therefore appears that the new position adopted by Arg 263 and the extra space provided by the Gly 79 in the *Nb*XIP1;1αL79G mutant are favorable for water permeability.

## Conclusion

Subtle changes, like the single point mutations L79G or I102H, are sufficient to allow for water permeability in *Nb*XIP1;1α. We conclude that *Nb*XIP1;1α is not likely to be occluded when heterologously expressed in the spheroplasts and that Thr 246 most probably resides at the H5^P^ position. Furthermore, most functional results can be explained from the models based on a combination of diameter and hydrophobicity of the ar/R filter. Our models suggest that a salt bridge between an acidic residue in a position corresponding to Asp 80 of *Nb*XIP1;1α directs the orientation of the arginine in the ar/R filter and provides a new way to tune the selectivity of AQPs. Published structures and models, together with sequence analysis, predict that the arginine is positioned in three different orientations in various XIPs. The engineered water permeable variants will facilitate further investigations of the structural and functional properties of *Nb*XIP1;1α in which e.g. the salt bridge, predicted residues in the LC^P^ position, oxidation state, phosphorylation and a putative gating may be studied.

## Additional files

Additional file 1: Table S1. Primers used in the PCR reactions. (PDF 45 kb)
Additional file 2: Figure S1. Expression of N-terminally His-tagged *Nb*XIP1;1α mutants in *P. pastoris*. Western blots showing the expression levels of N-terminally His-tagged *Nb*XIP1;1 mutants in *P. pastoris* X-33 clones. Blots were developed by enhanced chemiluminiscence in a Syngene PXi touch instrument. **a**. The first set of *Nb*XIP1;1 α mutants. *Nb*XIP1;1αst is an N-terminally truncated construct of *Nb*XIP1;1α used as control for the western blot. **b**. The second set of *Nb*XIP1;1α mutants. The control is 2 μg of purified *Nb*XIP1;1αwt. (PDF 207 kb)
Additional file 3: Table S2. Estimated protein amounts, rate constants and specific activities for the first set of *Nb*XIP1;1α mutants. (PDF 88 kb)
Additional file 4: Table S3. Estimated protein amounts, rate constants and specific activities for the second set of *Nb*XIP1;1α mutants. (PDF 87 kb)
Additional file 5: Table S4. Results of unpaired *t*-test for comparisons indicated in Fig. 5a. (PDF 44 kb)
Additional file 6: Table S5. Results of unpaired *t*-test for comparisons indicated in Fig. 5b. (PDF 45 kb)
Additional file 7: Figure S2. Cartoon representations of the homology models of *Nb*XIP1;1αwt and *Nb*XIP1;1α mutants. The pores in the models are shown in mesh representation from blue (widest) to red (narrowest). The HOLE program [38] was used to estimate the radius of the pore in the models. Starting from the top left to the bottom right; *Nb*XIP1;1αwt, *Nb*XIP1;1αL79G/I102H/**V242I** (mutant 1), *Nb*XIP1;1αL79G/I102H/**T246I** (mutant 2), *Nb*XIP1;1αL79G (mutant 3), *Nb*XIP1;1αI102H (mutant 4), *Nb*XIP1;1α**V242I** (mutant 5), *Nb*XIP1;1αL79G/I102H (mutant 6), *Nb*XIP1;1αI102H/**V242I** (mutant 7) and *Nb*XIP1;1αL79G/**V242I** (mutant 8), respectively. See also Fig. 7. (PDF 187 kb)

## Abbreviations

6xHis: Hexa-histidine
AQP1: Aquaporin 1
AQP8s: Aquaporin 8 s
AQPs: Aquaporins
ar/R: Aromatic/arginine
*At*TIP2;1: *Arabidopsis thaliana* TIP2;1
BMGY: Buffered glycerol-complex medium
BMMY: Buffered methanol-complex medium
ECL: Enhanced chemiluminiscence
EDTA: Ethylenediaminetetraacetic acid
FG-MD: Fragment-guided molecular dynamics simulation
GIPs: Glycerol facilitator-like intrinsic proteins
H2^P^: Helix 2 position
H5^P^: Helix 5 position
HE^P^: Helix E position
HIPs: Hybrid intrinsic proteins
His_10_-tag: Deca-histidine tag
I102H: Isoleucine 102 substituted for histidine
ICTAR: Isoleucine-cysteine-threonine-alanine-arginine
ICVAR: Isoleucine-cysteine-valine-alanine-arginine
IgG: Immunoglobulin G
I-TASSER: Iterative threading assembly refinement
L79G: Leucine 79 substituted for glycine
LC^P^: Loop C position
LE^P^: Loop E position
LGGC: Leucine-glycine-glycine-cysteine
MIPs: Major intrinsic proteins
NAMD: Nanoscale molecular dynamics
*Nb*XIP1;1α: *Nicotiana benthamiana* XIP1;1α
*Nb*XIP1;1β: *Nicotiana benthamiana* XIP1;1β
*Nb*XIP1;1: *Nicotiana benthamiana* XIP1;1
NIPs: Nodulin 26-like intrinsic proteins
NPA: Asparagine-proline-alanine aquaporin motif
NPARC: Asparagine-proline-alanine-arginine-cysteine
*Nt*XIP1;1: *Nicotiana tabacum* XIP1;1
OD_600_: Optical density at 600 nm
PCR: Polymerase chain reaction
PDB ID: Protein data bank identifier
PDB: Protein data bank
*Pf*AQP: *Plasmodium falciparum* aquaporin
PIPs: Plasma membrane intrinsic proteins
PMSF: Phenylmethylsulfonyl fluoride
pPICZB: *Pichia pastoris* vector for expression of proteins
*Pt*XIP1;1: *Populus trichocarpa* XIP1;1
PVDF: Polyvinylidene difluoride
SDS: Sodium dodecyl sulphate
SDS-PAGE: Sodium dodecyl sulphate - polyacrylamide gel electrophoresis
SIPs: Small basic intrinsic proteins
*So*PIP2;1: *Spinacia oleracea* PIP2;1
T246I: Threonine 246 substituted for isoleucine
TE-buffer: Tris–HCl EDTA buffer
TEV: Tobacco etch virus
TIP1s: Tonoplast intrinsic protein 1 s
TIP2-like: Tonoplast intrinsic protein 2-like
TIP3s: Tonoplast intrinsic protein 3 s
TIPs: Tonoplast intrinsic proteins
V242I: Valine 242 substituted for isoleucine
XIP1: X intrinsic protein 1
XIPs: X intrinsic proteins
YPD: Yeast extract peptone dextrose medium
YPDS: Yeast extract peptone dextrose medium with sorbitol
