# Supplementary material for: Single amino acid substitutions in the selectivity filter render NbXIP1;1α aquaporin water permeable
Source: BMC Plant Biol. 2017 Mar 9;17:61. doi: 10.1186/s12870-017-1009-3 (PMC5345251; doi:10.1186/s12870-017-1009-3)
Supplement: Additional file 1: Table S1. — Primers used in the PCR reactions. (PDF 45 kb) [file 12870_2017_1009_MOESM1_ESM.pdf]

Table S1. Primers used in the PCR reactions.

Primer name	Sequence
*Nb*XIP1;1αL79Gfwd	GGTTCTTGTTTTTATGGGTGACACAATAGTCATCTCC
*Nb*XIP1;1αL79Grev	GCCGAGCCTAGGAGCTCTCCCACT
*Nb*XIP1;1αI102Hfwd	TCATGTCAATTCTCCATGCAATTGTGATTAC
*Nb*XIP1;1αI102Hrev	TCAAATTTGGCATTTTCACATCACTTTCAAA
*Nb*XIP1;1αV242Ifwd	TTTGGGTCTGCTTATTTTCATCTCGACTAC
*Nb*XIP1;1αV242I rev	ACTATACCAACAATGGACAAGACAGTGACA
*Nb*XIP1;1αT246Ifwd	TGTGTTCATCTCGATTACAGTCACTGCGA
*Nb*XIP1;1αT246Irev	AGCAGACCCAAAACTATACCAACAATGGACA
*Nb*XIP1;1αloopCfwd	CCGGGCCCAAATGGGCCTATTAC
*Nb*XIP1;1αloopCrev	AGTAGAGCTAACTACTGCTTTAAGAGCTAGTGCACCTAA AATTG
*Nb*XIP1;1αL79G/I10 2H/V242IloopDfwd	GCTCTTGGACTTGTCACTGTCTTGTC
*Nb*XIP1;1αL79G/I10 2H/V242IloopDrev	TTGCCTATGATCATAAGCCATCCAAA
*Nb*XIP1;1αL79G/I10 2H/T246IloopDfwd	GTCACTGTCTTGTCCATTGTTGGTATAGTTT
*Nb*XIP1;1αL79G/I10 2H/T246IloopDrev	TTGCCTATGATCATAAGCCATCCAAA
