# Supplementary material for: Single amino acid substitutions in the selectivity filter render NbXIP1;1α aquaporin water permeable
Source: BMC Plant Biol. 2017 Mar 9;17:61. doi: 10.1186/s12870-017-1009-3 (PMC5345251; doi:10.1186/s12870-017-1009-3)
Supplement: Additional file 2: Figure S1. — Expression of N-terminally His-tagged NbXIP1;1α mutants in P. pastoris. Western blots showing the expression levels of N-terminally His-tagged NbXIP1;1 mutants in P. pastoris X-33 clones. Blots were developed by enhanced chemiluminiscence in a Syngene PXi touch instrument. a. The first set of NbXIP1;1 α mutants. NbXIP1;1αst is an N-terminally truncated construct of NbXIP1;1α used as control for the western blot. b. The second set of NbXIP1;1α mutants. The control is 2 μg of purified NbXIP1;1αwt. (PDF 207 kb) [file 12870_2017_1009_MOESM2_ESM.pdf]

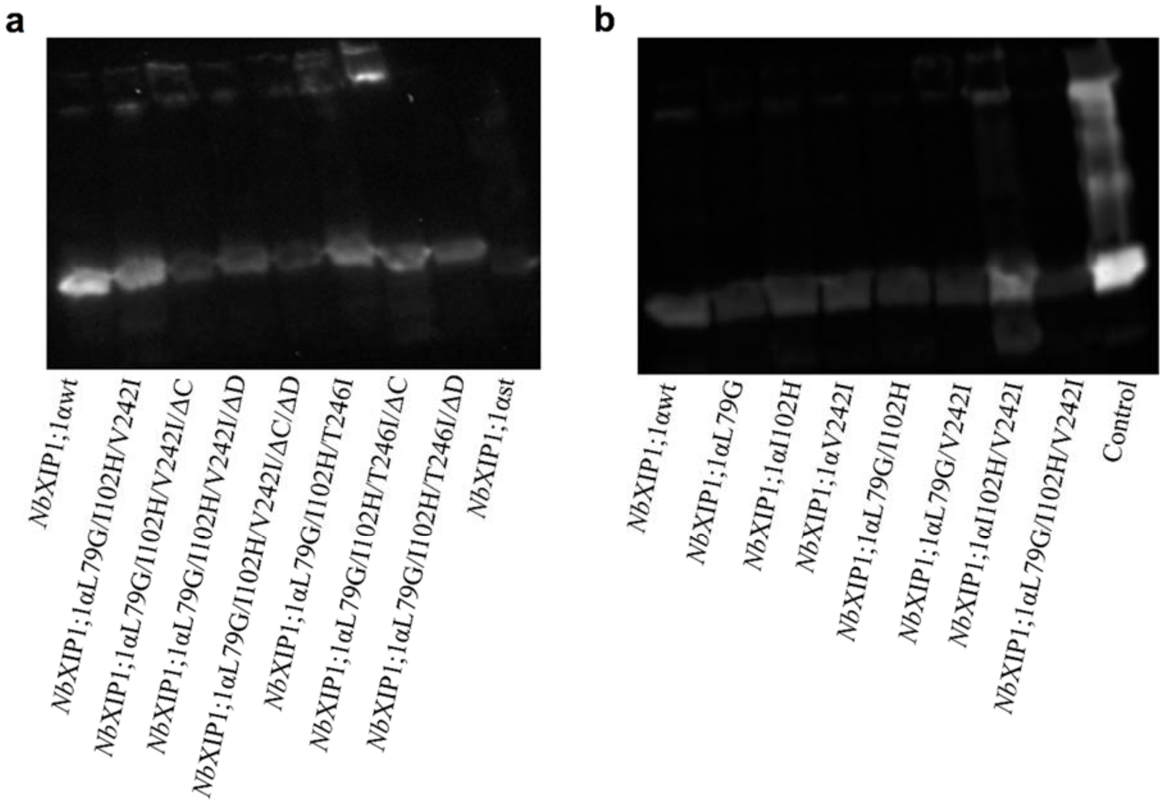

Fig. S1. Expression of N-terminally His-tagged *Nb*XIP1;1α mutants in *P. pastoris*. Western blots showing the expression levels of N-terminally His-tagged *Nb*XIP1;1 mutants in *P. pastoris* X-33 clones. Blots were developed by enhanced chemiluminiscence in a Syngene PXi touch instrument. **a**. The first set of *Nb*XIP1;1 α mutants. *Nb*XIP1;1αst is an N-terminally truncated construct of *Nb*XIP1;1α used as control for the western blot. **b**. The second set of *Nb*XIP1;1α mutants. The control is 2 μg of purified *Nb*XIP1;1αwt.
