# Supplementary material for: Single amino acid substitutions in the selectivity filter render NbXIP1;1α aquaporin water permeable
Source: BMC Plant Biol. 2017 Mar 9;17:61. doi: 10.1186/s12870-017-1009-3 (PMC5345251; doi:10.1186/s12870-017-1009-3)
Supplement: Additional file 3: Table S2. — Estimated protein amounts, rate constants and specific activities for the first set of NbXIP1;1α mutants. (PDF 88 kb) [file 12870_2017_1009_MOESM3_ESM.pdf]

Table S2. Estimated protein amounts, rate constants and specific activities for the first set of *Nb*XIP1;1α mutants.

*Nb*XIP1;1α mutant	Estimated protein amount (AU)	Background corrected rate constant (s^{-1})	Mean bkg corrected rate constant ± SD (s^{-1})	Specific activity (s^{-1} protein AU^{-1})	Mean specific activity ± SD (s^{-1} protein AU^{-1})
wt	2558	0.544	0.013 ± 0.487	0.000212	0.00000
		-0.093		-0.000036	±0.00019
		-0.413		-0.000161	
L79G/I102H	2305	4.228	3.325 ± 1.061	0.001833	0.00144
/V242I		2.157		0.000935	±0.00046
		3.591		0.001557	
L79G/I102H	949	0.865	0.526 ± 0.508	0.000911	0.00055
/V242I/ΔC		0.769		0.000810	±0.00054
		-0.058		-0.000060	
L79G/I102H	975	0.575	0.216 ± 0.372	0.000589	0.00022
/V242I/ΔD		0.243		0.000248	±0.00038
		-0.169		-0.000172	
L79G/I102H	775	0.047	-0.001 ± 0.581	0.000061	-0.00000
/V242I/ΔC/		0.556		0.000715	±0.00075
ΔD		-0.605		-0.000779	
L79G/I102H	1926	1.242	0.646 ± 0.535	0.000644	0.00034
/T246I		0.488		0.000253	±0.00028
		0.209		0.000108	
L79G/I102H	2262	0.101	-0.158 ± 0.606	0.000044	-0.00007
/T246I/ΔC		0.275		0.000121	±0.00027
		-0.850		-0.000375	
L79G/I102H	746	0.741	0.436 ± 0.535	0.000992	0.00058
/T246I/ΔD		0.750		0.001004	±0.00071
		-0.182		-0.000244	
