# Supplementary material for: Single amino acid substitutions in the selectivity filter render NbXIP1;1α aquaporin water permeable
Source: BMC Plant Biol. 2017 Mar 9;17:61. doi: 10.1186/s12870-017-1009-3 (PMC5345251; doi:10.1186/s12870-017-1009-3)
Supplement: Additional file 4: Table S3. — Estimated protein amounts, rate constants and specific activities for the second set of NbXIP1;1α mutants. (PDF 87 kb) [file 12870_2017_1009_MOESM4_ESM.pdf]

Table S3. Estimated protein amounts, rate constants and specific activities for the second set of *Nb*XIP1;1α mutants.

*Nb*XIP1;1α mutants	Estimated protein amount (AU)	Background corrected rate constant (s^{-1})	Mean background corrected rate constant ± SD (s^{-1})	Specific activity (s^{-1} protein AU^{-1})	Mean specific activity ± SD (s^{-1} protein AU^{-1})
wt	1628	-0.012	0.066 ± 0.173	-0.000007	0.00004
		0.265		0.000162	± 0.00011
		-0.053		-0.000032	
L79G	940	1.965	2.915 ± 0.823	0.002090	0.00310
		3.413		0.003630	± 0.00088
		3.367		0.003581	
I102H	1509	3.342	3.07 ± 0.255	0.002214	0.00203
		3.028		0.002006	± 0.00017
		2.838		0.001880	
V242I	1208	0.175	0.496 ± 0.386	0.000144	0.00041
		0.925		0.000765	± 0.00032
		0.389		0.000321	
L79G/I102H	2323	1.582	2.457 ± 1.046	0.000680	0.00106
		3.616		0.001556	± 0.00045
		2.174		0.000935	
L79G/V242I	867	3.132	3.316 ± 0.239	0.003610	0.00382
		3.586		0.004133	± 0.00028
		3.230		0.003723	
I102H/V242I	3859	5.578	7.938 ± 2.212	0.001445	0.00206
		8.273		0.002143	± 0.00057
		9.963		0.002581	
L79G/I102H/	555	3.782	4.324 ± 1.208	0.006813	0.00779
V242I		5.708		0.010282	± 0.00218
		3.483		0.006273	
