# Supplementary material for: Single amino acid substitutions in the selectivity filter render NbXIP1;1α aquaporin water permeable
Source: BMC Plant Biol. 2017 Mar 9;17:61. doi: 10.1186/s12870-017-1009-3 (PMC5345251; doi:10.1186/s12870-017-1009-3)
Supplement: Additional file 5: Table S4. — Results of unpaired t-test for comparisons indicated in Fig. 5a. (PDF 44 kb) [file 12870_2017_1009_MOESM5_ESM.pdf]

Table S4. Results of unpaired t-test for comparisons indicated in Fig. 5a.

Fig. 5a ID	Compared pair	*P*-value
a	*Nb*XIP1;1αwt & *Nb*XIP1;1αL79G/I102H/V242I	0.020
b	*Nb*XIP1;1αwt & *Nb*XIP1;1αL79G/I102H/T246I	0.173
c	*Nb*XIP1;1αL79G/I102H/V242I & *Nb*XIP1;1αL79G/I102H/T246I	0.033
