# Supplementary material for: Single amino acid substitutions in the selectivity filter render NbXIP1;1α aquaporin water permeable
Source: BMC Plant Biol. 2017 Mar 9;17:61. doi: 10.1186/s12870-017-1009-3 (PMC5345251; doi:10.1186/s12870-017-1009-3)
Supplement: Additional file 6: Table S5. — Results of unpaired t-test for comparisons indicated in Fig. 5b. (PDF 45 kb) [file 12870_2017_1009_MOESM6_ESM.pdf]

Table S5. Results of unpaired t-test for comparisons indicated in Fig. 5b.

Fig. 5b ID	Compared pair	*P*-value
a	*Nb*XIP1;1αwt & *Nb*XIP1;1αL79G	0.0248
b	*Nb*XIP1;1αwt & *Nb*XIP1;1αI102H	0.0002
c	*Nb*XIP1;1αwt & *Nb*XIP1;1αV242I	0.1743
d	*Nb*XIP1;1αL79G/I102H/V242I & *Nb*XIP1;1αL79G/I102H	0.0288
e	*Nb*XIP1;1αL79G/I102H/V242I & *Nb*XIP1;1αL79G/V242I	0.0851
f	*Nb*XIP1;1αL79G/I102H/V242I & *Nb*XIP1;1αI102H/V242I	0.0375
g	*Nb*XIP1;1αL79G/I102H/V242I & *Nb*XIP1;1αwt	0.0251
