# Supplementary material for: Single amino acid substitutions in the selectivity filter render NbXIP1;1α aquaporin water permeable
Source: BMC Plant Biol. 2017 Mar 9;17:61. doi: 10.1186/s12870-017-1009-3 (PMC5345251; doi:10.1186/s12870-017-1009-3)
Supplement: Additional file 7: Figure S2. — Cartoon representations of the homology models of NbXIP1;1αwt and NbXIP1;1α mutants. The pores in the models are shown in mesh representation from blue (widest) to red (narrowest). The HOLE program [38] was used to estimate the radius of the pore in the models. Starting from the top left to the bottom right; NbXIP1;1αwt, NbXIP1;1αL79G/I102H/V242I (mutant 1), NbXIP1;1αL79G/I102H/T246I (mutant 2), NbXIP1;1αL79G (mutant 3), NbXIP1;1αI102H (mutant 4), NbXIP1;1αV242I (mutant 5), NbXIP1;1αL79G/I102H (mutant 6), NbXIP1;1αI102H/V242I (mutant 7) and NbXIP1;1αL79G/V242I (mutant 8), respectively. See also Fig. 7. (PDF 187 kb) [file 12870_2017_1009_MOESM7_ESM.pdf]

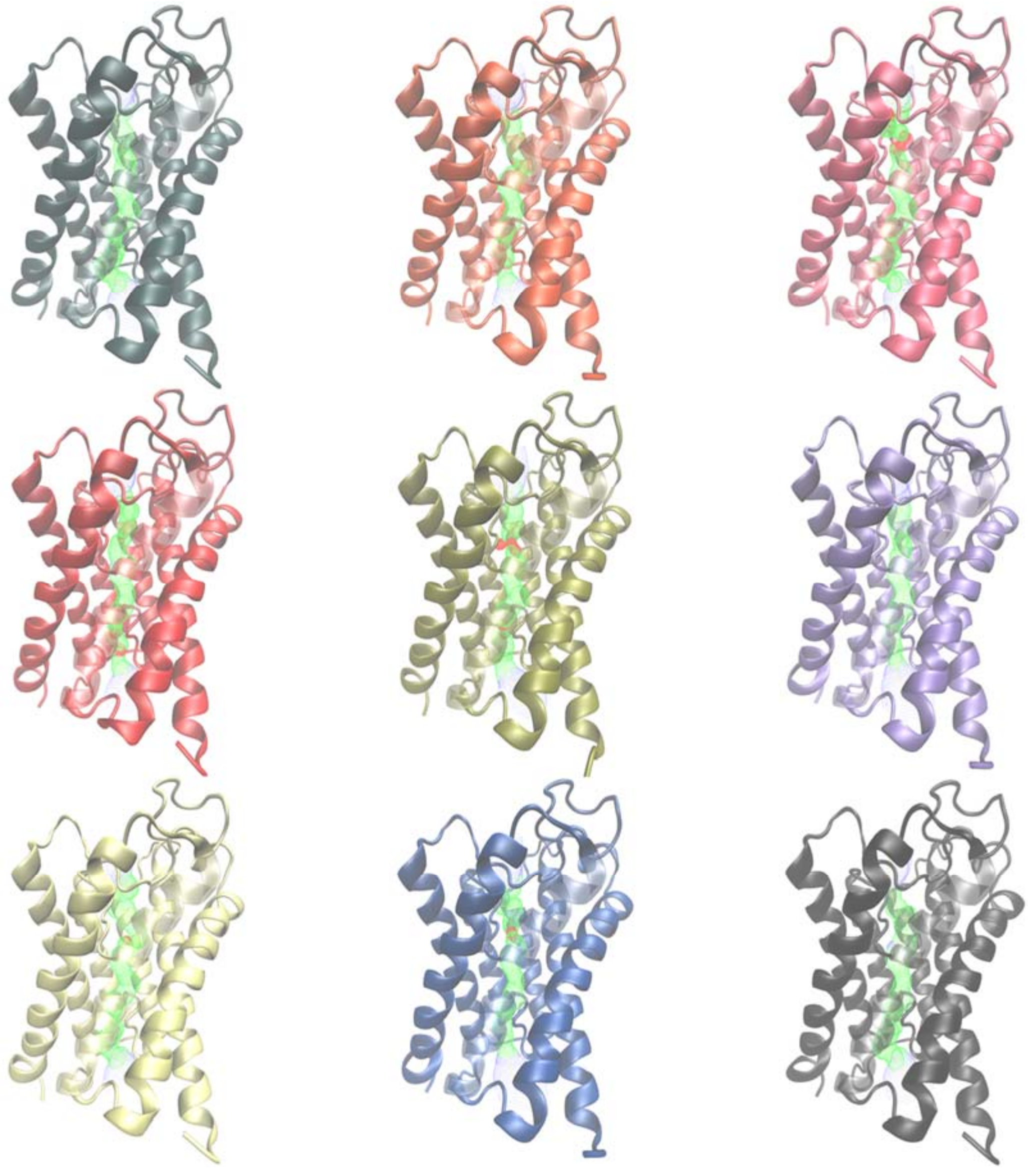

Fig. S2. Cartoon representations of the homology models of *Nb*XIP1;1αwt and *Nb*XIP1;1α mutants. The pores in the models are shown in mesh representation from blue (widest) to red (narrowest). The HOLE program [36] was used to estimate the radius of the pore in the models. Starting from the top left to the bottom right; *Nb*XIP1;1αwt, *Nb*XIP1;1αL79G/I102H/V242I (mutant 1), *Nb*XIP1;1αL79G/I102H/T246I (mutant 2), *Nb*XIP1;1αL79G (mutant 3), *Nb*XIP1;1αI102H (mutant 4), *Nb*XIP1;1αV242I (mutant 5), *Nb*XIP1;1αL79G/I102H (mutant 6), *Nb*XIP1;1αI102H/V242I (mutant 7) and *Nb*XIP1;1αL79G/V242I (mutant 8), respectively. See also Fig. 7.
